# TBC1D1 interacting proteins, VPS13A and VPS13C, regulate GLUT4 homeostasis in C2C12 myotubes

**DOI:** 10.1038/s41598-020-74661-1

**Published:** 2020-10-21

**Authors:** Sharon C. Hook, Alexandra Chadt, Kate J. Heesom, Shosei Kishida, Hadi Al-Hasani, Jeremy M. Tavaré, Elaine C. Thomas

**Affiliations:** 1 School of Biochemistry, Biomedical Sciences Building, University of Bristol, University Walk, Bristol, BS8 1TD UK; 2 Institute of Clinical Biochemistry and Pathobiochemistry, German Diabetes Center, Leibniz Center for Diabetes Research at Heinrich Heine University, Medical Faculty, Düsseldorf, Germany; 3 German Center for Diabetes Research (DZD), München-Neuherberg, Germany; 4 Department of Biochemistry and Genetics, Kagoshima University Graduate School of Medical and Dental Sciences, Kagoshima, Japan

**Keywords:** Proteomics, Phosphoproteins, Intracellular signalling peptides and proteins, Diabetes, Membrane trafficking, Protein translocation

## Abstract

Proteins involved in the spaciotemporal regulation of GLUT4 trafficking represent potential therapeutic targets for the treatment of insulin resistance and type 2 diabetes. A key regulator of insulin- and exercise-stimulated glucose uptake and GLUT4 trafficking is TBC1D1. This study aimed to identify proteins that regulate GLUT4 trafficking and homeostasis via TBC1D1. Using an unbiased quantitative proteomics approach, we identified proteins that interact with TBC1D1 in C2C12 myotubes including VPS13A and VPS13C, the Rab binding proteins EHBP1L1 and MICAL1, and the calcium pump SERCA1. These proteins associate with TBC1D1 via its phosphotyrosine binding (PTB) domains and their interactions with TBC1D1 were unaffected by AMPK activation, distinguishing them from the AMPK regulated interaction between TBC1D1 and AMPKα1 complexes. Depletion of VPS13A or VPS13C caused a post-transcriptional increase in cellular GLUT4 protein and enhanced cell surface GLUT4 levels in response to AMPK activation. The phenomenon was specific to GLUT4 because other recycling proteins were unaffected. Our results provide further support for a role of the TBC1D1 PTB domains as a scaffold for a range of Rab regulators, and also the VPS13 family of proteins which have been previously linked to fasting glycaemic traits and insulin resistance in genome wide association studies.

## Introduction

Skeletal muscle is a major sink for plasma glucose in the body, with insulin- and exercise-stimulated contraction being its principle physiological stimuli^1^. These stimuli activate distinct signalling pathways, both culminating in the mobilisation or translocation of intracellular stores of GLUT4 glucose transporters, sequestered within GLUT4 storage vesicles (GSV), to the cell surface to facilitate removal of glucose from the blood. Impaired glucose disposal, caused by defective plasma membrane translocation of GLUT4, has been linked to insulin-resistance and type 2 diabetes^2,3^. However, individuals with these diseases have preserved exercise-stimulated glucose uptake due to activation of insulin-independent signalling pathways^4^. As such, proteins involved in insulin-independent glucose uptake present as attractive therapeutic targets for treatment of insulin-resistance and type II diabetes.

TBC1D1 is a Rab-GTPase-Activating Protein (Rab-GAP), closely related to AS160 (TBC1D4)^5^, that regulates glucose uptake in muscle cells^6^. These Rab-GAP proteins maintain Rab proteins in the inactive GDP-bound form and hence function to prevent GLUT4 translocation in the absence of stimuli^5,7^. In support of this, cells depleted of, or overexpressing, TBC1D1 exhibit increased and decreased plasma membrane GLUT4, respectively^5,8,9^.

TBC1D1 contains multiple phosphorylation sites for the Ser/Thr kinases Akt and AMPK^5,10^. Mutational studies have demonstrated that phosphorylation of key residues, namely Thr^596^ and Ser^237^ by Akt and AMPK respectively, are important in enabling GLUT4 translocation^11,12^, indicating that TBC1D1 is at a key regulatory position linking signalling with the trafficking steps to facilitate glucose uptake. Consequently, TBC1D1 is considered a convergence point between insulin- and contractile-stimulated signalling^13,14^.

Several lines of evidence support the importance of TBC1D1 in regulating whole body energy homeostasis. TBC1D1 is associated with obesity-related traits and type 2 diabetes in both humans^15–19^ and mice^20–22^. In addition, variation in *TBC1D1* is associated with growth- and obesity-related traits in pigs, chickens and rabbits^23–27^. Genetic knockout models of TBC1D1, in both mice and rats, have increased fatty acid oxidation, indicative of altered metabolic substrate utilisation^20,28,29^.

Both TBC1D1 and AS160 possess two phosphotyrosine-binding (PTB) domains, a central calmodulin-binding domain and a C-terminal Rab-GAP domain^15^. Whilst the function of the TBC1D1 PTB domains are poorly understood, evidence from AS160^30,31^ and TBC1D1^32–34^ studies suggest regulatory and signalling roles. Indeed, TBC1D1 PTB domain binding of (i) the GLUT4 storage resident protein, IRAP, is disrupted by phosphorylation^35^ and (ii) AMPKα1 heterotrimeric complexes enhance phosphorylation of its key regulatory site, Ser^237^ ^33^. Consequently, binding partners may represent novel effectors of TBC1D1 function, thereby influencing the subcellular distribution of GLUT4 and as such may serve as targets by which the pharmaceutical industry could develop a new generation of diabetes drugs.

Here we reveal additional proteins that bind to the PTB domains of TBC1D1, identified using Stable Isotope Labelling by Amino acids in Cell culture (SILAC), with this study aimed to further characterise the interactome and establish if these proteins play a role in regulating GLUT4 biology. Using this method, we previously identified AMPK itself as forming a stable interaction with the PTB domains of TBC1D1^33^. In the current study we show that the TBC1D1 PTB domains stably associate with several Rab regulatory proteins including MICAL1 and EHBP1L1, the calcium pump SERCA1 and VPS13A and VPS13C. Unlike with AMPKα1-containing complexes, the interaction of these novel proteins with TBC1D1 was not regulated by AMPK activation. We further identified VPS13A and VPS13C as regulators of stimulated GLUT4 translocation to the cell surface.

## Results

### TBC1D1 stably associates with proteins involved in trafficking

**Figure 1:**
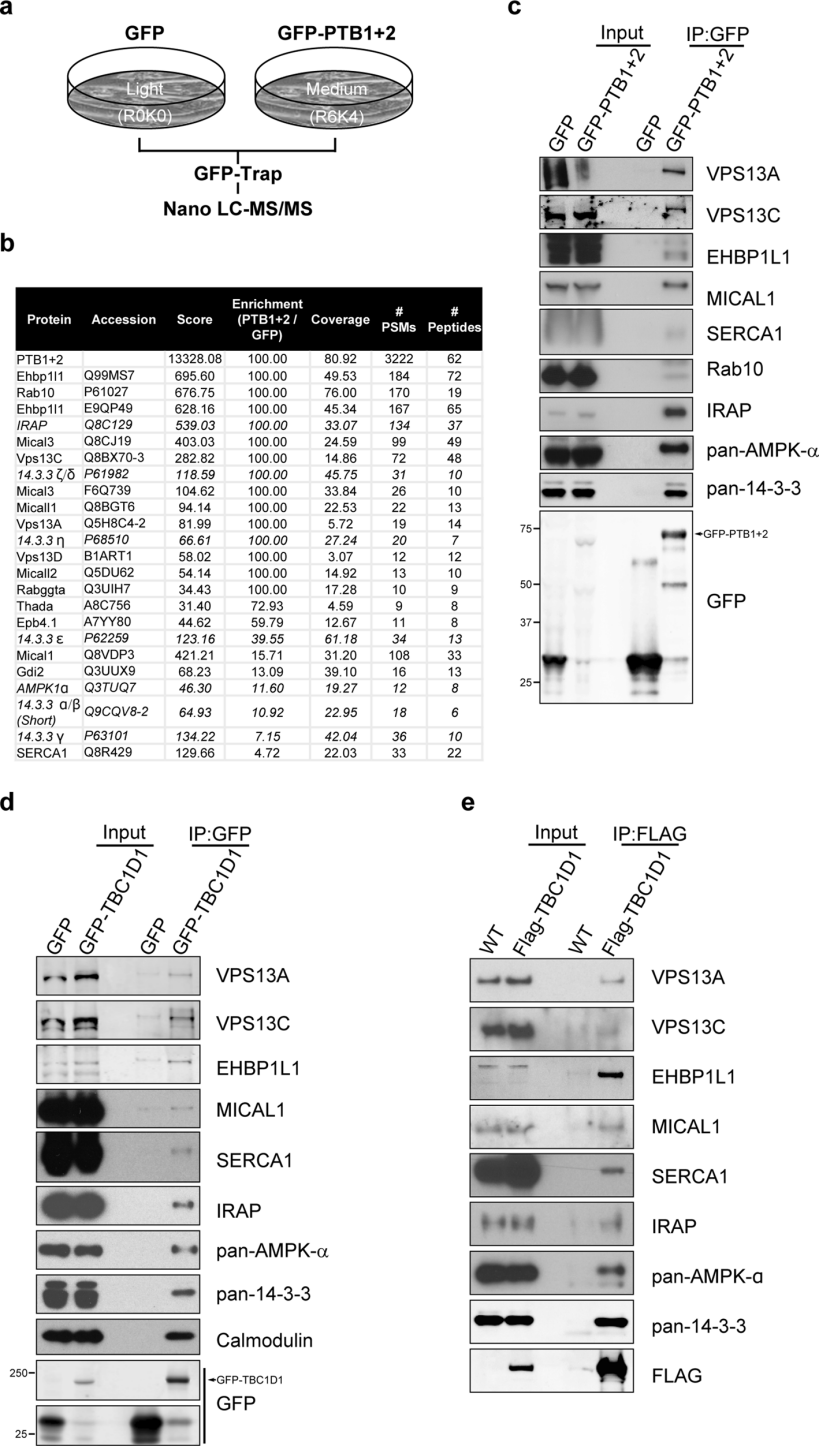
Identification and validation of the TBC1D1 PTB protein interactome. (**a**) Schematic of the SILAC experiment setup used to identify proteins interacting with the PTB1 and PTB2 domains (amino acids 1–381) of TBC1D1 in differentiated C2C12 myotubes. (**b**) Table of proteomics output filtered for a score > 30, enrichment > 4 and #PSMs > 8 (raw dataset is in Supplementary Table S1). Shown in italics are the previous reported hits^33^. Score: the sum of the Xcorr values for the peptides matched to that protein (a high score generally signifies a high protein abundance and a high confidence in the detection and quantification by the software). Enrichment: Ratio of the quantification values of the medium (GFP-PTB1 + 2) and light (GFP) quantification channels. A ratio of 100 suggests that the protein was not detected at all in the light (GFP) channel. Coverage: percentage of the protein sequence covered by identified peptides. # PSMs (peptide spectrum matches): total number of identified peptide sequences (includes those redundantly identified). # Peptides: total number of unique identified peptides. (**c**) Western blot validation of proteomics results. GFP-trap precipitates from C2C12 myotube extracts, lentivirally transduced to express either GFP or GFP-PTB1 + 2 blotted for the proteins indicated. (**d**) Lysates from differentiated C2C12 myotubes lentivirally transduced with GFP or GFP-TBC1D1 subjected to GFP-trap and western blotting for the proteins indicated. (**e**) Homogenised quadriceps lysates from either wild type (WT) mice or mice expressing 3xFLAG-TBC1D1 were immunoprecipitated with an anti-FLAG antibody and resulting complexes analysed. Representative blots of n = 4 independent experiments.

In our previous study we reported AMPKα1, as a subset of proteins, identified to interact with the TBC1D1 PTB domains through SILAC-based quantitative proteomics on GFP-trap based immunoprecipitations from C2C12 myotubes stably expressing a GFP-tagged construct consisting of the two PTB domains of TBC1D1 (GFP-PTB1 + 2; Fig. 1a)^33^. Here, shown in Fig. 1b and Supplementary Table S1, we reveal the full dataset generated in our previous study of proteins enriched, by 5- to 100-fold, in the GFP-PTB1 + 2 precipitates compared to the GFP control. This included known interactors of the TBC1D1 PTB domains; several isoforms of 14–3-3 (the α/β, γ, ε, ζ/δ and η) proteins and IRAP (insulin-responsive aminopeptidase)^30,35–38^, as we previously reported^33^.

As shown in Fig. 1b, we additionally observed > 15-fold enrichment in the GFP-PTB1 + 2 precipitates of three members of the VPS13 family (VPS13A, VPS13C and VPS13D), three MICAL protein family members (MICAL1, MICAL2 and MICAL3), EHBP1L1 and Rab10. We also observed a 4.7-fold enrichment of the calcium pump, SERCA1.

EHBP1L1, a Rab8a effector involved in vesicle formation^39^, is a paralog of EHBP1 a Rab10 effector protein which has been implicated in regulating GLUT4 trafficking to the plasma membrane (PM) in adipocytes^40^. Both EHBP1L1 and MICAL proteins possess calponin-homology domains that facilitate actin binding, and coiled-coil regions involved in protein interactions^39,41^. VPS13A and VPS13C, members of the highly conserved VPS13 family^42^, are lipid binding proteins^43^ that have been shown to be associated with several organelles^43–46^, regulators of cytoskeleton dynamics^47–49^ and vesicular trafficking^47,50^.

The interactome was validated by western blotting a selection of proteins including VPS13A, VPS13C, EHBP1L1, MICAL1 and SERCA1 (Fig. 1c) which were additionally confirmed to associate with full-length GFP-tagged TBC1D1 expressed in C2C12 myotubes (Fig. 1d) and to a FLAG-tagged TBC1D1 transgene expressed in mouse quadriceps muscles (Fig. 1e). These results indicate that TBC1D1 associates with a constellation of proteins known to facilitate trafficking, with an affinity to its PTB domains that is sufficiently high to survive immunoprecipitation and extensive washing. Rab10 did not appear to bind to the full-length protein with sufficient affinity to survive immunoprecipitation.

### AICAR stimulation has no effect on the novel TBC1D1 PTB interactome

**Figure 2:**
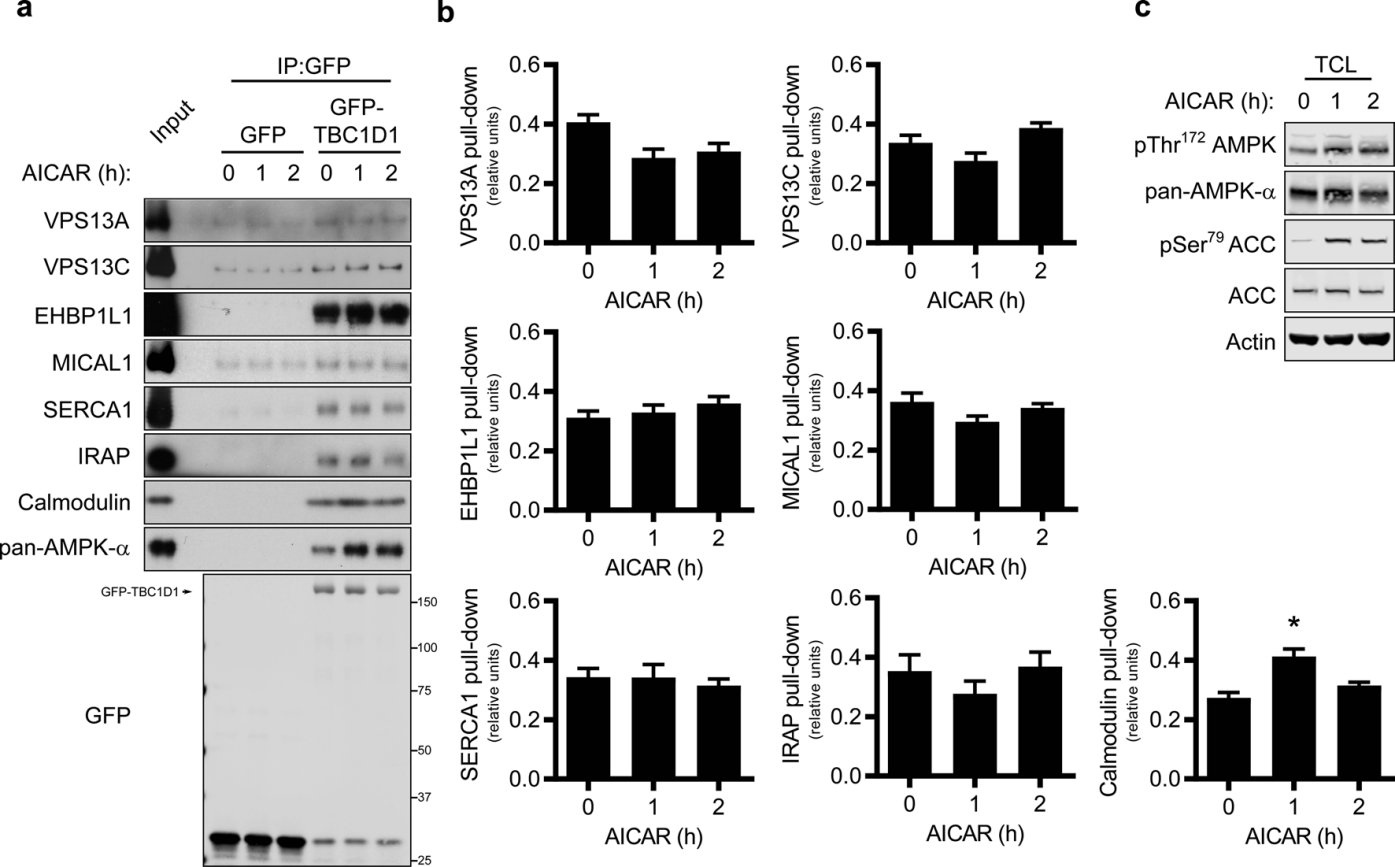
AICAR stimulation has little effect on the TBC1D1 interactome. (**a**) Western blot analysis of GFP-trap precipitates from differentiated C2C12 myotubes lentivirally transduced with GFP or GFP-TBC1D1, serum starved for 4–5 h, prior to addition of vehicle or AICAR (2 mM) for the times indicated. (**b**) Quantitation of data shown in (**a**). Protein pull-down was normalised to GFP-TBC1D1 precipitated and then expressed as relative values. as Mean ± SEM; 3–6 independent experiments; repeated measures one-way ANOVA Dunnett’s post-test * p < 0.05 *cf* Basal. (**c**) Total cell lysates (TCL) from (**a**) analysed for total and phosphorylated proteins.

Given the regulatory role of TBC1D1 in AMPK-mediated glucose uptake in skeletal muscle^11,20^, we next examined whether AMPK activation affected the association of the validated interacting proteins. Unlike our previously demonstrated increased association of α1-subunit containing AMPK complexes and 14-3-3 proteins with TBC1D1^33^, stimulation with the AMPK activator AICAR did not result in any significant alteration in the association of VPS13A, VPS13C, MICAL1, SERCA1, EHBP1L1 or IRAP with TBC1D1 in C2C12 myotubes (Fig. 2a,b). This was despite enhanced AMPK and ACC phosphorylation which confirmed that AMPK was indeed activated under these conditions (Fig. 2c). We did, however, observe a transient increase in the interaction of calmodulin with the full-length TBC1D1 protein (Fig. 2a,b), presumably via the putative calmodulin-binding domain in the central core of TBC1D1^15^ since calmodulin is not observed in our PTB1 + 2 alone interactome (Fig. 1). Taken together, this data suggests that the novel TBC1D1 PTB-domain interactome we have validated is not subject to general regulation by AMPK.

### Depletion of VPS13A and VPS13C increases total and stimulated surface levels of GLUT4

**Figure 3:**
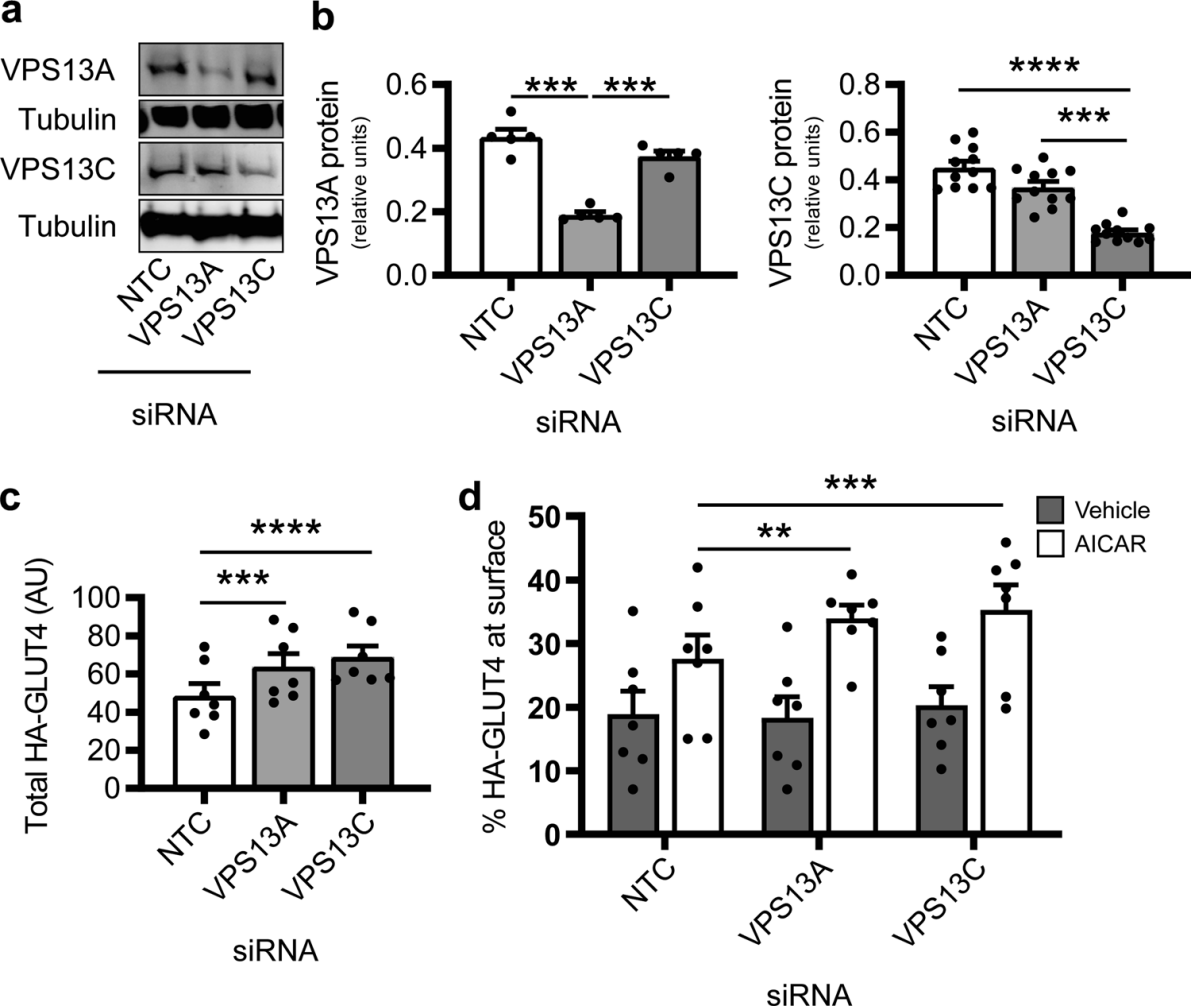
Depletion of either VPS13A or VPS13C increases stimulated GLUT4 translocation and total GLUT4 levels. (**a**) Differentiating C2C12 myoblasts were transiently transfected with non-targeting control (NTC) siRNA, or siRNA pool against either VPS13A or VPS13C. Endogenous protein levels were examined by western blotting 72 h after transfection. (**b**) Quantitation of data shown in (**a**) normalised to tubulin and then shown as relative values Mean ± SEM; VPS13A and VPS13C protein from 5 or 10 independent experiments respectively; repeated measures one-way ANOVA Tukey post-test ** p < 0.01, *** p < 0.001, **** p < 0.0001. (**c**) Total protein levels of HA-GLUT4 as measured via high-throughput plate-based translocation assay, as in (**d**), expressed in arbitrary units (AU). Mean ± SEM; 7 independent experiments; repeated measures one-way ANOVA Dunnett’s post-test ** p < 0.01, **** p < 0.0001 *cf* NTC. (**d**) C2C12 myotubes transiently transfected with indicated siRNA were serum starved for 4 h in media containing 0.2% BSA prior to addition of vehicle (black bars) or stimulation with AICAR (2 mM, 45 min; white bars). Surface HA-GLUT4 was measured in non-permeabilised cells as described in the methods. Shown is the percentage of HA-GLUT4 at the PM relative to the total level of HA-GLUT4. Mean ± SEM; 7 independent experiments; repeated measures two-way ANOVA Dunnett’s post-test ** p < 0.01, *** p < 0.001 *cf* NTC.

We next sought to identify if novel TBC1D1 interacting proteins have a role in regulating GLUT4 biology. We investigated the role of VPS13A and VPS13C in GLUT4 translocation to the plasma membrane, as the *VPS13C* gene locus has been linked to type 2 diabetes risk and glycaemic traits^51–59^. To do this we used isoform-specific siRNA pools to knockdown either VPS13A or VPS13C in differentiated C2C12 myotubes stably expressing a GLUT4 construct possessing an exofacial HA-tag, which allowed measurement of total and cell surface exposed GLUT4. These siRNA pools produced a 55.4% and 58.2% reduction in VPS13A and VPS13C proteins, respectively (Fig. 3a,b). Importantly, siRNA knockdown of VPS13A had no effect on protein levels of VPS13C, and vice versa.

We utilised the AMPK activator, AICAR, to stimulate translocation of HA-GLUT4 to the cell surface in a high-throughput plate-based translocation assay used to detect surface and total cellular levels of HA-GLUT4 (Fig. 3c,d). siRNA knockdown of either VPS13A or VPS13C increased the total levels of HA-GLUT4 present in the cell compared to non-targeting control siRNA (Fig. 3c). This was accompanied by an enhancement in the proportion of cellular HA-GLUT4 at the cell surface in the presence of AICAR, but not in its absence (Fig. 3d). As such this resulted in VPS13A or VPS13C depleted myotubes with approximately two-fold higher level of HA-GLUT4 present at the cell surface upon AICAR stimulation compared to control myotubes (Fig. S1). Together this data indicates that members of the VPS13 family of proteins are involved in regulation of subcellular distribution of GLUT4 in myotubes.

### Total and surface levels of other recycling plasma membrane proteins are unaffected by siRNA-mediated knockdown of VPS13A and VPS13C

We next investigated whether the effect of VPS13A and VPS13C depletion on total and surface levels of GLUT4 was selective for this glucose transporter, or impacted on a range of other membrane proteins that similarly cycle between the plasma membrane and cell interior. These proteins included were the insulin-regulated glucose transporter GLUT1^60^ and the fatty acid transporter CD36^61,62^ as well as the constitutively recycling proteins EGFR, N-cadherin and the transferrin receptor^63,64^.

**Figure 4:**
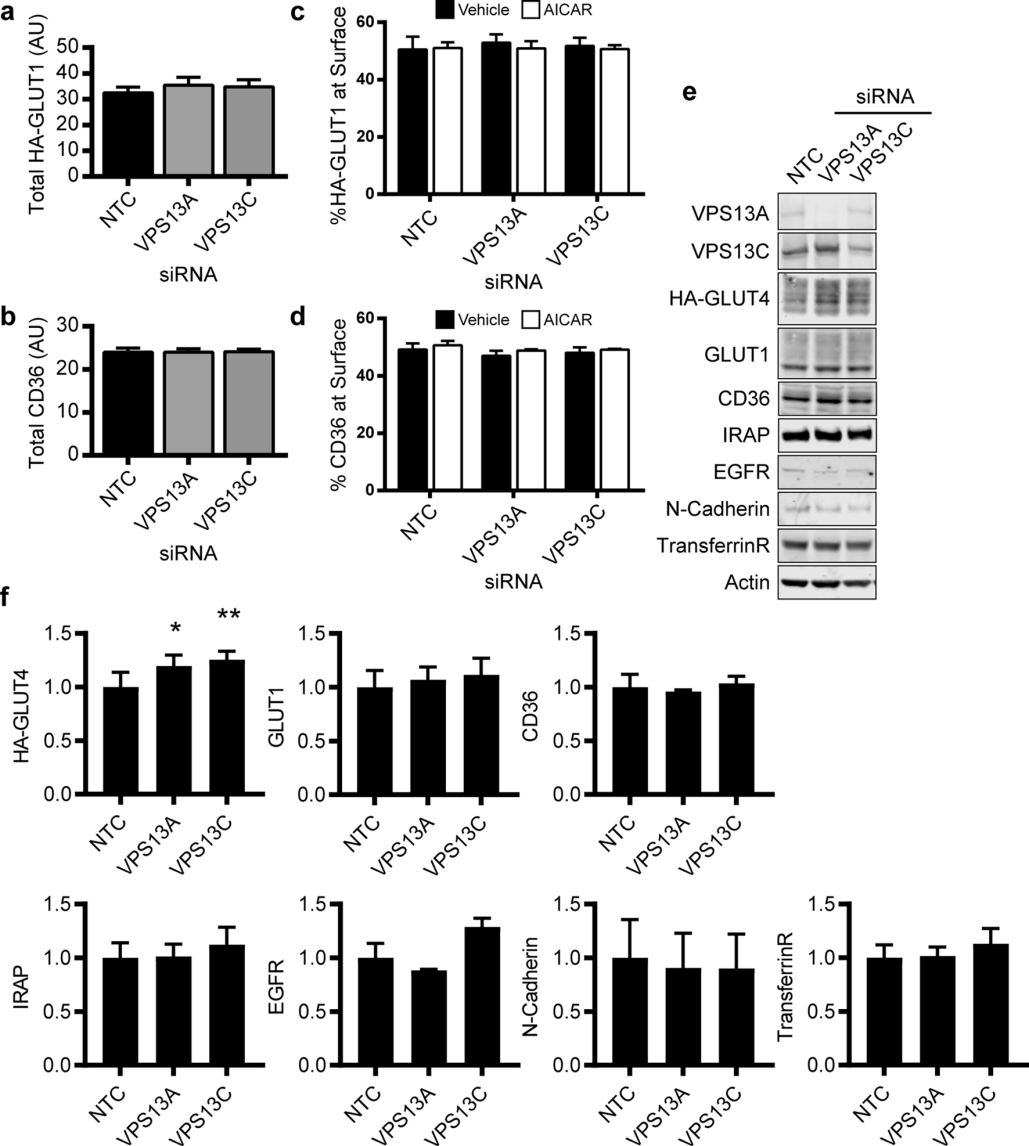
Depletion of VPS13A and VPS13C is without effect on other cell surface proteins. C2C12 myotubes transiently transfected with indicated siRNA were serum starved for 4 h in media containing 0.2% BSA prior to stimulation with AICAR (2 mM, 45 min). Total cellular levels of (**a**) HA-GLUT1 or (**b**) CD36 expressed in arbitrary units (AU). Mean ± SEM; 4 independent experiments. Surface (**c**) HA-GLUT1 or (**d**) CD36 were measured as described in the methods. Shown is the percentage of HA-GLUT1 or CD36 at the cell surface relative to the total cellular level of HA-GLUT1 or CD36 respectively. Mean ± SEM; 4 independent experiments. (**e**) C2C12 myotubes transiently transfected with indicated siRNA were harvested and subjected to western blotting for the proteins indicated. (**f**) Quantitation of fluorescence-based western blot data shown in (**e**). Data were normalised to the actin loading control and expressed as a fold by comparison to the NTC condition mean value. Mean ± SEM; 3–4 independent experiments; one-way ANOVA Dunnett’s post-test * p < 0.05, ** p < 0.01 *cf* NTC.

We used a high-throughput plate-based assay to detect stably expressed HA-tagged GLUT1 and endogenous CD36 in C2C12 myotubes (Fig. 4a–d). In contrast to HA-GLUT4, the total cellular levels of HA-GLUT1 and CD36 were unaffected by siRNA knockdown of VPS13A or VPS13C (Fig. 4a,b). AICAR stimulation did not alter the percentage of either HA-GLUT1 (Fig. 4c) or CD36 (Fig. 4d) at the cell surface in the C2C12 myotubes, as anticipated, and siRNA mediated knockdown of VPS13A or VPS13C had no effect on the surface levels of these proteins.

The levels of expression of GLUT4, GLUT1, CD36, IRAP, EGFR, N-cadherin and the transferrin receptor were then examined by western blotting lysates from control, VPS13A or VPS13C depleted myotubes (Fig. 4e,f). Increased levels of HA-GLUT4 protein were confirmed in VPS13A or VPS13C depleted myotubes. In contrast, total protein levels of GLUT1, CD36, IRAP, EGFR, N-cadherin and the transferrin receptor were not significantly different in myotubes depleted of VPS13A or VPS13C versus the control. Taken together this data suggests VPS13A and VPS13C regulate GLUT4 protein levels but not that of the other recycling proteins we examined.

### HA-GLUT4 endocytosis, gene transcription and AMPK signalling are unaffected in myotubes depleted of VPS13A or VPS13C

**Figure 5:**
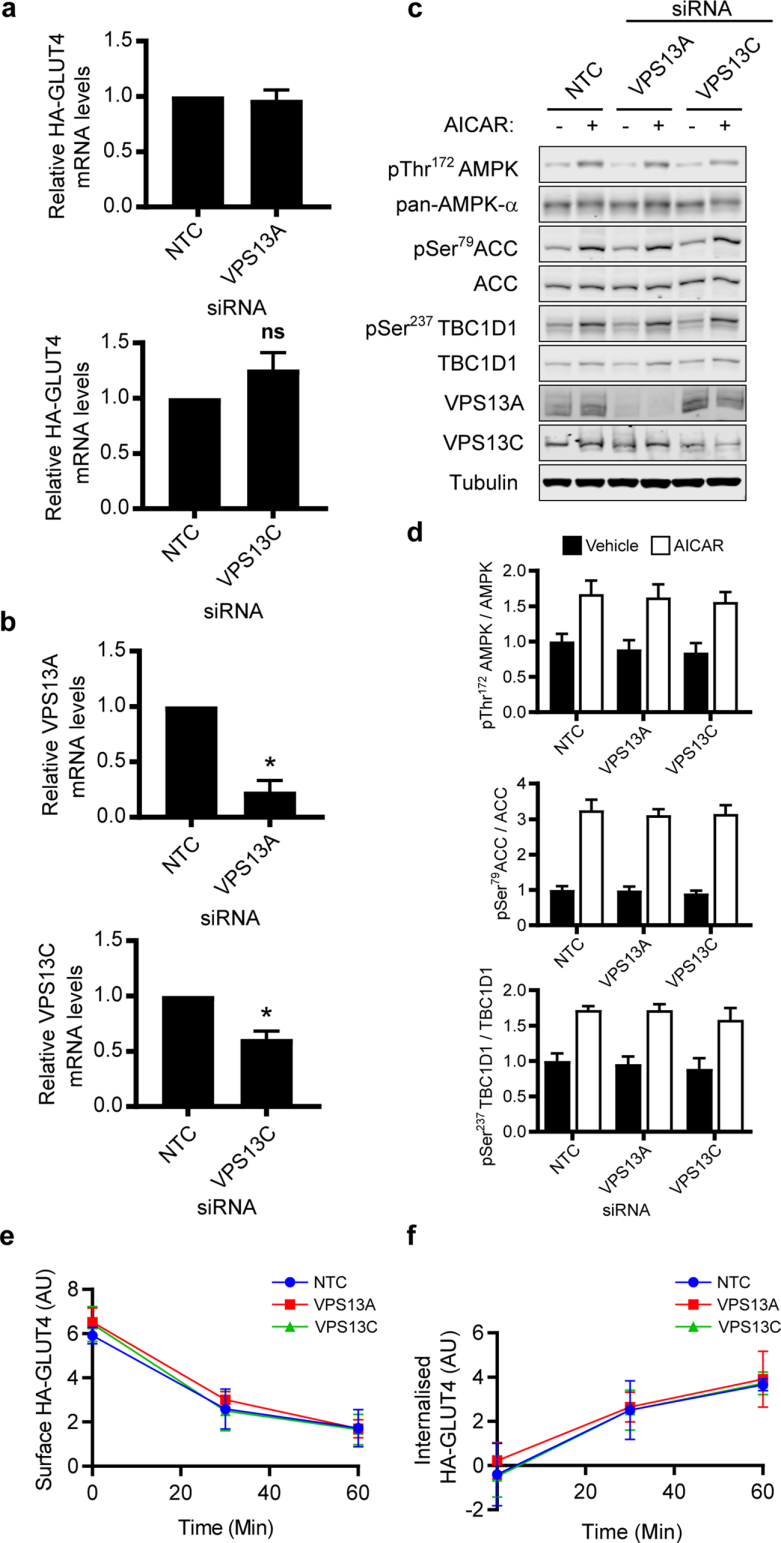
HA-GLUT4 endocytosis, gene transcription and AICAR-stimulated signalling are unaffected in myotubes depleted of VPS13A or VPS13C. Differentiating C2C12 myoblasts were transiently transfected with non-targeting control (NTC) siRNA or siRNA pool against either VPS13A or VPS13C. (**a**) RNA was extracted 72 h post transfection and relative mRNA transcript levels of HA-GLUT4 as determined by qRT-PCR. (**b**) Confirmation of siRNA mediated knock-down of VPS13A and VPS13C via qRT-PCR. Mean ± SEM; 3 independent experiments; Welch’s t-test * p < 0.05. (**c**) Protein levels were examined by western blotting 72 h after transfection following serum starvation for 4 h in media containing 0.2% BSA prior to addition of vehicle (black bars) or AICAR (2 mM, 45 min; white bars). (**d**) Quantitation of fluorescence-based western blot data shown in (**c**). AMPK phosphorylation normalised to total AMPKα expression; ACC phosphorylation normalised to total ACC expression; TBC1D1 phosphorylation normalised to total TBC1D1 expression. All displayed as a fold over mean NTC basal phosphorylation. Mean ± SEM; 4 independent experiments; no significant difference *cf* NTC basal or *cf* NTC AICAR two-way ANOVA Dunnett’s post-test. (**e**,**f**) Differentiating C2C12 myoblasts were transiently transfected with non-targeting control (NTC) siRNA (blue circles), or siRNA pool against either VPS13A (red squares) or VPS13C (green triangles). Myotubes were then serum starved for 4 h in media containing 0.2% BSA prior to AICAR addition (2 mM, 45 min), subsequent labelling with anti-HA antibody and processing as described in the materials and methods. (**e**) Detected bound antibody to HA-GLUT4 at the cell surface over time. (**f**) Detected internalised antibody bound to HA-GLUT4 over time. Mean ± SEM; 4 independent experiments; no significant difference *cf* NTC two-way ANOVA Dunnett’s post-test.

To determine whether the effect of VPS13A and VPS13C knockdown was related to transcription of the GLUT4 gene, we examined HA-GLUT4 mRNA transcript levels (Fig. 5a) and found these to be unchanged in myotubes subjected to VPS13A or VPS13C siRNA knockdown (Fig. 5b). We next assessed whether the increased presentation of HA-GLUT4 at the cell surface, upon VPS13A or VPS13C knockdown, could be attributed to an alteration in AICAR-stimulated signalling. Stimulation of C2C12 myotubes with AICAR resulted in the significant increase in phosphorylation of AMPK, in addition to the AMPK substrates TBC1D1 and ACC in both control and VPS13A or VPS13C depleted myotubes with no significant difference observed in the extent of phosphorylation upon VPS13A or VPS13C depletion (Fig. 5c,d). We then investigated whether the increased expression and surface levels of GLUT4 in VPS13A- or VPS13C-depleted GLUT4 were the result of changes in GLUT4 endocyosis using a high-throughput antibody-based GLUT4 internalisation assay after AICAR stimulation (2 mM 45 min). As shown in Fig. 5e,f, a time-dependent decrease in cell surface HA-GLUT4 was observed concomitant with an increase in internalised HA-GLUT4 during GLUT4 endocytosis, but this process after 60 min was unaffected by depletion of either VPS13A or VPS13C.

## Discussion

In the present study we reveal additional novel validated interacting proteins of TBC1D1, a key regulator of GLUT4 translocation and glucose uptake in skeletal muscle. The results add significant credibility to the concept that the PTB domains of TBC1D1 function as important protein–protein interaction modules. Furthermore, we demonstrate that members of the VPS13 family, VPS13A and VPS13C, are important in maintaining GLUT4 homeostasis in myotubes.

We and others have previously demonstrated that the PTB domains of TBC1D1 bind to 14–3-3 proteins^33,36–38^, IRAP^30,33,35^, AMPK complexes containing α1 but not α2 catalytic subunits^33^ and APPL2^65^. Using a differentiated in vitro muscle cell model and intact mouse quadriceps muscle, we extend this repertoire of validated binding proteins to include VPS13A, VPS13C, EHBP1L1, MICAL1 and SERCA1. These proteins all interacted with the isolated PTB domains of TBC1D1, as well as the full-length proteins. We further demonstrate that Rab10 binds to the isolated PTB domains, but not to the full-length protein, at least with sufficient affinity to survive the immunoprecipitation procedure.

We obtained evidence for the additional presence of MICAL2, MICAL3, VPS13D, Rabggta, Thada, Epb4.1 and Gdi2 in GFP-PTB1 + 2 immunoprecipitates. All were greater than tenfold enriched versus GFP control, however we have not formally validated these interactions using western blotting. Interestingly, we also found that calmodulin interacts with full-length TBC1D1 but consider this is likely to be via the central calmodulin-binding domain of the protein because we did not find any peptides derived from calmodulin in the GFP-PTB1 + 2 interactome (Fig. 1a). TBC1D1 has a similar domain structure and ∼50% identical primary sequences with AS160^5^ and it will be interesting to examine whether the proteins that bind TBC1D1 also interact with AS160.

In contrast to our previous observations with 14-3-3 and AMPK^33^, and a recent report with IRAP^35^, pharmacological activation of AMPK in C2C12 myotubes did not significantly affect the interaction of TBC1D1 with VPS13A, VPS13C, EHBP1L1, SERCA1, MICAL1 or IRAP (Fig. 2). The AMPK phosphorylation site at Ser^237^ within PTB2 provides part of the basis by which AMPKα1 containing complexes bind to TBC1D1. Intriguingly, a recent study showed IRAP binding TBC1D1 was disrupted upon phosphorylation of TBC1D1 by AMPK or Akt in an in vitro system^35^ which may suggest stoichiometry of phosphorylation and/or cellular context, including other interacting proteins, play a role in regulating this interaction. Future studies will be required to establish if other stimuli of GLUT4 translocation modulate the TBC1D1 interactome, particularly insulin to which C2C12 cells are poorly responsive, and muscle contraction in intact muscles.

In further exploring the role of TBC1D1 interactors, we focussed specifically on both VPS13C, because polymorphisms near its gene locus have previously been associated with type 2 diabetes risk and glycaemic traits^51–59^, and VPS13A, as it is the closest VPS13C paralogue of the VPS13 proteins^42^. Moreover, while VPS13D was also identified as an interactor (Fig. 1b) we were unable to validate it via western blotting with the limited commercial antibodies available. VPS13 proteins have been reported to bind to a PxP motif on adaptor proteins in endosomes, vacuole membranes and mitochondria of yeast^66,67^. Whether this sequence-dependent recruitment is important in the binding of mammalian VPS13 proteins to adaptor proteins remains to be established, however, interestingly, both PTB1 and PTB2 domains of TBC1D1 possess PxP motifs in surface exposed regions of our homology models^68^. These may serve to recruit VPS13 proteins to membrane compartments such as GSVs, via TBC1D1.

Using siRNA-mediated knockdown in C2C12 myotubes, we found that depletion of either VPS13A or VPS13C resulted in increased levels of GLUT4 protein (Figs. 3d and 4f), but not of the mRNA transcript (Fig. 5a). This suggests that the effect occurs post-transcriptionally. The phenomenon appears to be specific for GLUT4 as other proteins that cycle between the cell surface and intracellular locations and engage a range of trafficking and sorting machineries (GLUT1, IRAP, transferrin receptors, CD36, EGFR and N-cadherin) were unaffected, as determined by (i) high-throughput plate-based assay on intact myotubes (Fig. 4c,d) and (ii) western blotting of myotube lysates (Fig. 4e,f). A lack of effect on IRAP expression and trafficking is, therefore, surprising given that it shares much of the same trafficking itinerary and machinery as GLUT4. While this manuscript was in preparation, a study by Munoz-Braceras et al.^69^ reported in HeLa cells VPS13A interacts with the lysosomal protein Rab7A and VPS13A depletion diminished lysosomal degradation. Among several cargo investigated EGFR degradation was reduced, which contrasts with our results; it is currently unclear how VPS13A may play a role in lysosomal function. AICAR stimulated signalling, including phosphorylation of Ser^237^ on TBC1D1, remained intact in myotubes depleted of VPS13A or VPS13C (Fig. 5c,d). Moreover, the increased presence of GLUT4 at the cell surface upon AICAR stimulation was not due to reduced GLUT4 endocytosis as the rate of internalisation from the surface was indistinguishable between control and VPS13A or VPS13C depleted myotubes (Fig. 5e,f). Together this suggests the elevated cell surface GLUT4 levels results from an altered exocytosis profile. Future studies will need to investigate the kinetics of GLUT4 exocytosis in addition to establishing if there is increased sequestered GLUT4 in GSVs available for translocation upon AICAR stimulation in VPS13A and VPS13C depleted cells.

In contrast to our observations with VPS13A and VPS13C depletion, the expression of the GLUT4 protein is known to be reduced in skeletal muscles of mouse models of TBC1D1 deficiency^20–22,70^ a phenomenon which has been attributed to missorting and enhanced post-translational degradation of the protein^71^. Future investigations will be required to determine the dependence of the interaction of TBC1D1 with VPS13A and VPS13C in regulating stimulated GLUT4 translocation and expression levels as well as confirming the distinct step(s) in the pathway modulated by VPS13A/C depletion.

The precise biological functions of VPS13A and VPS13C are currently unclear as they have been ascribed to be involved in several biological pathways. Recent evidence has shown VPS13A and VPS13C transport glycerolipids, such as phosphatidylcholine and phosphatidylinositol, between membrane compartments via a hydrophobic tunnel and hence act as a tether between the ER and LE/Lysosome (VPS13C), mitochondria (VPS13A) and lipid droplets (VPS13A/C)^43^. As lipid transporters, VPS13A and VPS13C have the capacity to control the lipid homeostasis of different membranes^43^ and hence the capacity to alter the composition of different compartments. Little is known about the lipid profile of the specialised GLUT4 compartment, although it is known that changes in plasma membrane lipids, evoked in response to insulin, promote GSV fusion with the plasma membrane^72^. An intriguing possibility is that through modulating the lipid profile of the GSV, the VPS13 proteins could alter the fusion competency of the GSV. Given VPS13A or VPS13C depletion enhances AICAR-stimulated GLUT4 translocation, it could suggest maintenance of GSV lipid composition by these proteins acts to limit fusion competency.

The function of TBC1D1 has been best characterised in the context of skeletal muscle, however emerging evidence supports a role for TBC1D1 in other tissues including the pancreas^73–75^. On the basis that ablation of TBC1D1 results in a mild insulin secretion phenotype^74,76^ and genome wide association studies have linked the *VPS13C* locus to beta cell impairment^51,56,57,77,78^, a role for the VPS13 proteins and other TBC1D1 interactors in the insulin secreting beta cell should be explored.

Intriguingly, several of the other proteins we found in the TBC1D1 PTB1 + 2 precipitates are regulators of Rab proteins. This includes MICAL1 which binds Rab10^79^ and EHBP1L1 which binds Rab8^39^, both of which we validated as interactors by western blotting (Fig. 1). Two other proteins were identified in the TBC1D1 interactome, Rabggta and Gdi2. While we did not formally validate these by western blotting, they were 100- and 13-fold enriched in the PTB1 + 2 precipitates versus controls (Fig. 1b). Rabggta is a geranylgeranyl transferase type-2 that adds hydrophobic geranylgeranyl moieties to the C-terminus of many of the Rab proteins and is critical for their delivery to the correct intracellular target membrane compartments^80^. Gdi2 is a Rab GDP-dissociation inhibitor that binds geranylgeranylated Rab proteins^81^ and is required for the delivery and removal of Rab proteins from their target membranes^82^.

Rab2A, 8A, 8B, 10, 14, 28, 32, 33B and 34 are known substrates for the GTPase Activating Protein (GAP) domain located at the C-terminus of TBC1D1^5,7,83^. The association of an array of Rab regulatory proteins with the N-terminal PTB domains of TBC1D1 could provide a mechanism for carefully orchestrating the binding and activity of an array of cognate Rab proteins bound to the C-terminal GAP domain at different stages of the GLUT4 trafficking cycle through the cell. The interaction of TBC1D1 with the calcium pump SERCA1 is also intriguing and will require further investigation, most especially given the fact that muscle contraction is an important physiological regulator of muscle glucose uptake.

In summary, our results provide increasingly convincing proof that the PTB domains of TBC1D1 serve as a scaffold that is capable of recruiting a constellation of proteins and regulators involved in GLUT4 vesicle trafficking. Understanding the precise nature of these protein interactions represents an important next step in validating whether these have the potential to serve as therapeutic targets for the treatment of insulin-resistance and type 2 diabetes.

## Materials and methods

### Antibodies and reagents

Unless otherwise stated all reagents were from Sigma-Aldrich. Primary antibodies against pan-AMPK-α (#2532), pAMPK Thr^172^ (#2531), ACC (#3876), pACC Ser^79^ (#3661), EGFR (#2232), IRAP (#3808), N-cadherin (#14215), Rab10 (#8127S) and rabbit FLAG (#2368) were from Cell Signaling Technology. CD36 (ab124515), Calmodulin (ab45689), GLUT1 (ab115730) and SERCA1 (ab129104) were from Abcam. From Santa Cruz Biotechnology Inc were antibodies against pan-14-3-3 (sc-629) and transferrin receptor (sc-65882). Other antibodies were EHBP1L1 (#PAB15793; Abnova), GFP (118144600001; Roche), HA.11 16B12 (901503; Biolegend), MICAL1 (14818–1-AP; proteintech), pTBC1D1 Ser^237^ (#07-2268; Merck-Millipore), VPS13C (HPA043356; Atlas Antibody), β actin (A1978), Tubulin (T9026) and mouse FLAG (F1804). VPS13A antibody was produced as previously described^84^. Secondary antibodies used were conjugated with an AlexaFluor (Invitrogen) or HRP (Cell Signaling Technology or Jackson ImmunoResearch). AICAR was from Tocris.

### Mouse model

Transgenic MCK-3xFLAG-*Tbc1d1* mice with muscle-specific overexpression of *Tbc1d1* were generated as previously described^33^ and kept in accordance with the National Institutes of Health guidelines for the care and use of laboratory animals, and all experiments were approved by the Ethics Committee of the State Ministry of Agriculture, Nutrition and Forestry (State of North Rhine-Westphalia, Germany). Male mice between 10 and 14 weeks were sacrificed by cervical dislocation, quadricep muscle dissected and directly snap-frozen in liquid nitrogen.

### Molecular biology

Constructs for lentiviral expression of full-length TBC1D1 and TBC1D1 PTB1 + 2, amino acids 1–381 based on secondary structure prediction^68^, were cloned as previously described^33^ into pXLG3. Also cloned into pXLG3 were HA-GLUT4 and HA-GLUT1, both with a HA-tag in the first exofacial loop, from pQB125-HA-GLUT4^85^ and pCDNA3-HA-GLUT1^86^ respectively.

### Cell culture

C2C12 (American Type Culture Collection) were cultured in DMEM supplemented with 10% (v/v) FBS (Invitrogen), 50 IU/ml penicillin, 50 μg/ml streptomycin and 2 mM glutamine. C2C12 myoblasts were differentiated into myotubes as previously described^87^. HEK293T cells were used to produce lentiviral particles which were subsequently added to C2C12 myoblasts to generate stably transduced cell lines. Transduction efficiency was 80–85% as determined by immunofluorescence.

### SILAC interactome analysis

SILAC samples were generated in our previous study^33^, which described only a subset of the interacting proteins, with this current study we reveal the full dataset. Briefly, transduced C2C12 myoblasts were expanded in R0K0 or R6K4 isotopically labelled DMEM (Dundee Cell Products) supplemented with 10% (v/v) dialysed FBS (Dundee Cell Products) for at least six passages prior to differentiation to achieve full labeling. Myoblasts were then differentiated in R0K0 or R6K4 isotopically labelled DMEM supplemented with 2% (v/v) dialysed horse serum. Myotubes were harvested in precipitation buffer 50 mM Tris–HCl pH 7.4, 50 mM NaCl, 1 mM EDTA, 1 mM EGTA, 10% glycerol, 0.5% NP40, 1 mM DTT, 50 mM NaF, 5 mM Na_4_P_2_O_7_, 1 mM Na_3_VO_4_, 0.5 mM PMSF, Calbiochem protease inhibitor cocktail), lysates incubated (4 °C, 1 h) with GFP-trap beads (ChromoTek), precipitates washed four times with precipitation buffer and samples pooled prior to eluting, separation on NuPAGE 4–12% precast gel (Invitrogen) and Nano LC–MS/MS on an Orbitrap Velos mass spectrometer (Thermo Fisher Scientific). Mass spectrometric detection and quantification was performed as previously published^88^.

### Immunoprecipitation and western blot analysis

Transduced C2C12 myotubes were harvested in precipitation buffer and GFP-trap performed as per above. For mouse muscle immunoprecipitations, protein-G-coated agarose beads pre-conjugated with anti-FLAG antibody were blocked in precipitation buffer containing 1% (w/v) BSA prior to incubation overnight at 4 °C with homogenised quadriceps muscle (4 mg) or precipitation buffer and subsequent washing, as previously described^33^. For analysis of total cell lysates, myotubes were harvested in ice-cold lysis buffer (50 mM HEPES pH 7.4, 150 mM NaCl, 1% Triton-X-100, 1 mM Na_3_VO_4_, 30 mM NaF, 10 mM Na_4_P_2_O_7_, 10 mM EDTA, 0.5 mM PMSF, Calbiochem protease inhibitor cocktail), protein content determined by BCA assay (Thermo Fisher Scientific) and equal amounts resolved by SDS-PAGE. Standard western blot procedures were performed. Fluorescently labelled secondary antibodies were detected via a LI-COR Odyssey infrared imaging system and HRP-conjugated secondary antibodies via ECL. Samples were normalized to loading control, total protein or to precipitated protein as indicated in the legends. Subsequently western blot values were normalised to the sum of all data points in a replicate (normalisation by summation)^89^ (Figs. 2b, 3b) or normalised to the mean of the control (Figs. 4f, 5d).

### siRNA knockdown

For siRNA knockdown, after 48 h in differentiation media differentiating C2C12 cells were transfected with siRNA using DharmaFECT (Dharmacon) according to manufacturer’s instructions replacing back to differentiation media after 4 h. The siRNA from GE Healthcare used in this study were: ON-TARGETplus non-targeting pool (D-001810-10), ON-TARGETplus mouse VPS13A (M-066653-00) and ON-TARGETplus mouse VPS13C (L-053177-00). Myotubes were assayed 72 h after transfection.

### GLUT4 translocation and antibody uptake assays

For the GLUT translocation assays, C2C12 myoblasts or transduced C2C12 myoblasts expressing either HA-GLUT4 or HA-GLUT1 were plated in triplicate at 7000 cells per well of a 96 well plate coated with 1% gelatin and differentiated as per above. 68 h after siRNA transfection myotubes were serum starved for 4 h in media containing 0.2% (w/v) BSA with vehicle or AICAR (2 mM) added for times indicated, fixed in 4% (v/v) paraformaldehyde (Electron Microscope Services) and quenched with 0.15 M glycine. To detect surface levels of HA-GLUT4 or HA-GLUT1, cells were firstly incubated with blocking solution (1% (w/v) BSA/3% (v/v) goat serum/PBS) and then with anti-HA antibody (1:500) in 1% (w/v) BSA/PBS. To detect total cellular levels of HA-GLUT4 or HA-GLUT1 cells were incubated with blocking solution containing 0.2% (w/v) saponin and antibody solution containing 0.2% (w/v) saponin. All cells were then washed thrice in PBS, incubated in blocking solution and then in anti-mouse secondary antibody coupled to AlexaFluor700 (1:200) in 1% (w/v) BSA/PBS and finally washed thrice in PBS. Signal was detected by a LI-COR Odyssey infrared imaging system in plate mode. To account for any non-specific antibody labelling, the signal detected in cells not expressing HA-GLUT4 protein was subtracted. CD36 translocation assays were performed the same except using antibodies to detect CD36 (1:500) and an anti-rabbit secondary antibody coupled to AlexaFluor800.

For the antibody uptake assays, cells were plated, differentiated, transfected and allowed to basal as per the translocation assay, prior to stimulation with AICAR (2 mM 45 min). To label cell surface HA-GLUT4, cells were firstly washed thrice in ice-cold DMEM, then incubated on ice with anti-HA antibody (1:200) in DMEM for 30 min and washed thrice in ice-cold DMEM to remove unbound antibody. Cells were either immediately fixed with 4% (v/v) paraformaldehyde at 37 °C or returned to 37 °C/5% CO_2_ for 30 or 60 min to allow uptake of the antibody to occur prior to fixation. To label antibody bound at the cell surface, washed cells were incubated in blocking solution then with anti-mouse secondary antibody coupled to AlexaFluor700 (1:500) in 1% (w/v) BSA/PBS. After washing, control wells or wells to detect internalised antibody were incubated with blocking solution with or without 0.2% (w/v) saponin respectively, followed by incubation with anti-mouse secondary antibody coupled to AlexaFluor800 (1:500) in 1% (w/v) BSA/PBS with or without 0.2% (w/v) saponin respectively. Following washing, all wells were incubated with blocking solution containing 0.2% (w/v) saponin, washed and fluorescence at 700 nm and 800 nm was detected using a LI-COR Odyssey infrared imaging system in plate mode. To account for any non-specific background surface labelling, the signal detected on cells not expressing HA-GLUT4 protein was subtracted. To account for any non-specific background surface labelling when detecting internalized antibody, the signal detected from non-permeabilised wells, at the equivalent timepoint, was also subtracted.

### qRT-PCR

RNeasy kit (Qiagen) was used to extract total RNA from C2C12 myotubes. qRT-PCR was performed using the SuperScript III Platinum SYBR Green One-Step qRT-PCR kit (Invitrogen). All samples were normalised to levels of GAPDH mRNA. Primers used for amplification of fragments are listed in Table S2.

### Statistics

Statistical analyses were performed using GraphPad Prism 8.4.3 software. To account for variability between experiments repeated measures ANOVA was used to “block” treatment data with the control data as appropriate^90^. As indicated in the figure legends, for comparisons of two groups Welch’s t-test was used; for multiple comparisons the following statistical tests were used: repeated measures one-way ANOVA followed by Dunnett’s post-test or Tukey post-test, or two-way ANOVA followed by Tukey post-test or Dunnett’s post-test.

## Supplementary information

Supplementary file1

Supplementary file2
