# Supplementary material for: TBC1D1 interacting proteins, VPS13A and VPS13C, regulate GLUT4 homeostasis in C2C12 myotubes
Source: Sci Rep. 2020 Oct 21;10:17953. doi: 10.1038/s41598-020-74661-1 (PMC7578007; doi:10.1038/s41598-020-74661-1)

TBC1D1 INTERACTING PROTEINS, VPS13A AND VPS13C, REGULATE GLUT4 HOMEOSTASIS IN C2C12 MYOTUBES

Sharon C. Hook[1], Alexandra Chadt[2,3], Kate J. Heesom[1], Shosei Kishida[4], Hadi Al-Hasani[2,3], Jeremy M. Tavaré[1], Elaine C. Thomas[1]*

SUPPLEMENTARY INFORMATION

Figure S1. Total surface GLUT4 levels upon depletion of either Vps13A or Vps13C.

C2C12 myotubes transiently transfected with indicated siRNA were serum starved for 4 h in media containing 0.2% BSA) prior to addition of vehicle (black bars) or stimulation with AICAR (2mM, 45 min; white bars). Surface HA-GLUT4 was measured as described in the methods. Data is from Fig 3C displayed here as amount of HA-GLUT4 at the cell surface normalised to the mean surface HA-GLUT4 in the NTC basal condition. Mean ± SEM; 7 independent experiments; repeated measures two-way ANOVA Dunnett post-test, **** $p<0.0001$ *cf* NTC.

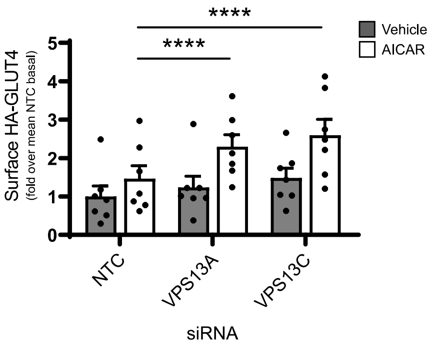

Table S2. List of qRT-PCR primers used in this study

Gene	Forward (5’-3’)	Reverse
GAPDH	atgtttgtgatgggtgtgaa	atgccaaagttgtcatggat
Vps13A	tcattctccttacgcctgct	caggtgaagcaattcccaat
Vps13C	cagctaaagctggggatctg	cagggaccacaagcaggtat
HA-GLUT4	cttcgagacagcaggggtag	aggagcagagccacagtcat

Unprocessed Western blots

Figure 1c

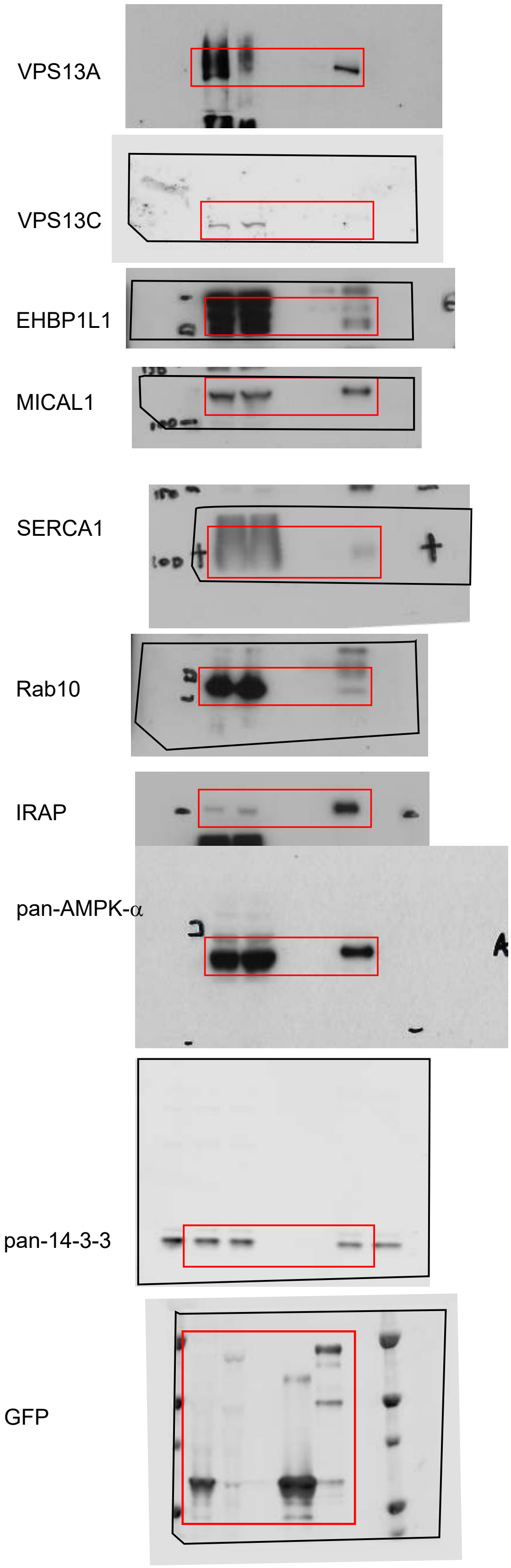

Figure 1d

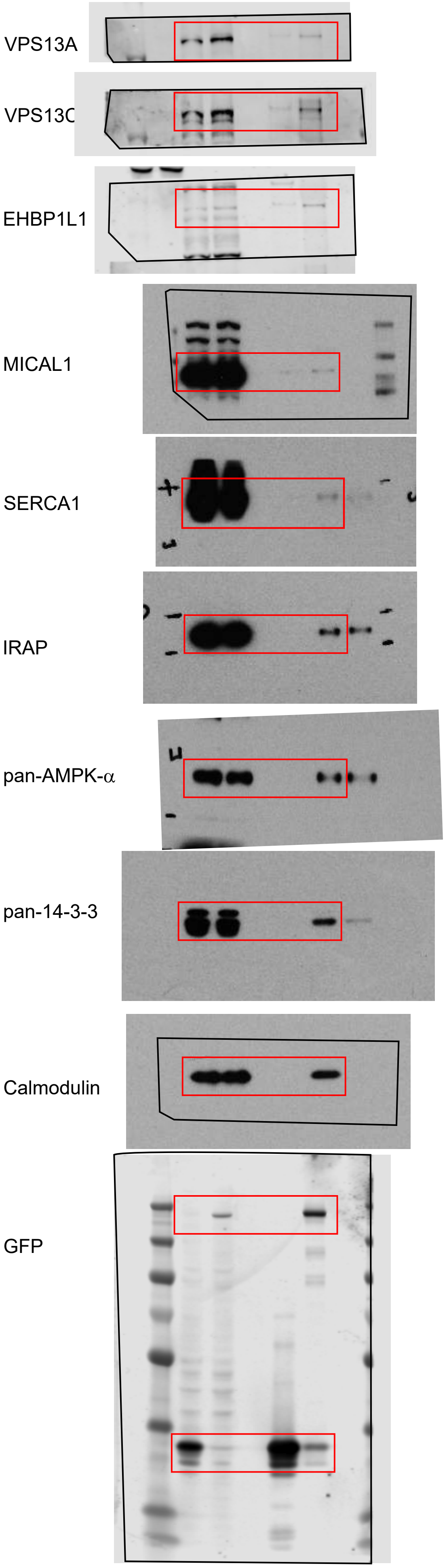

Figure 1e

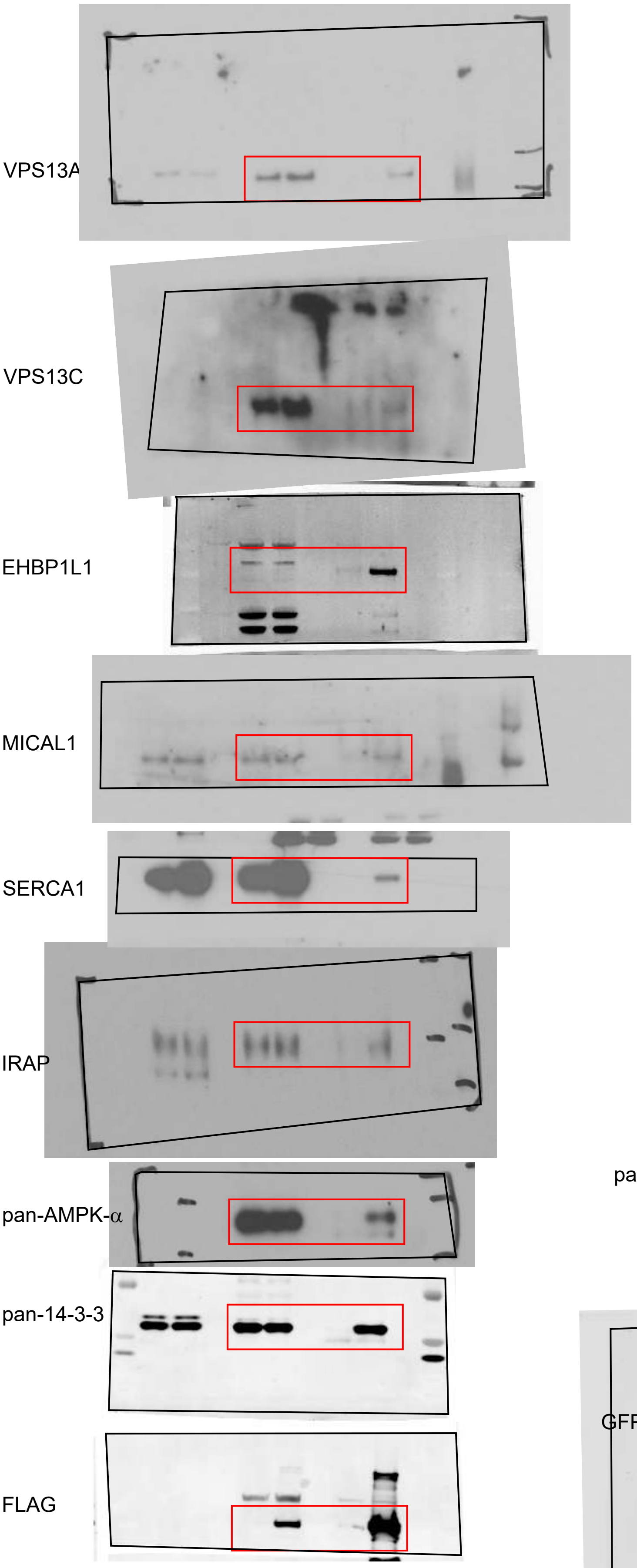

Figure 2a

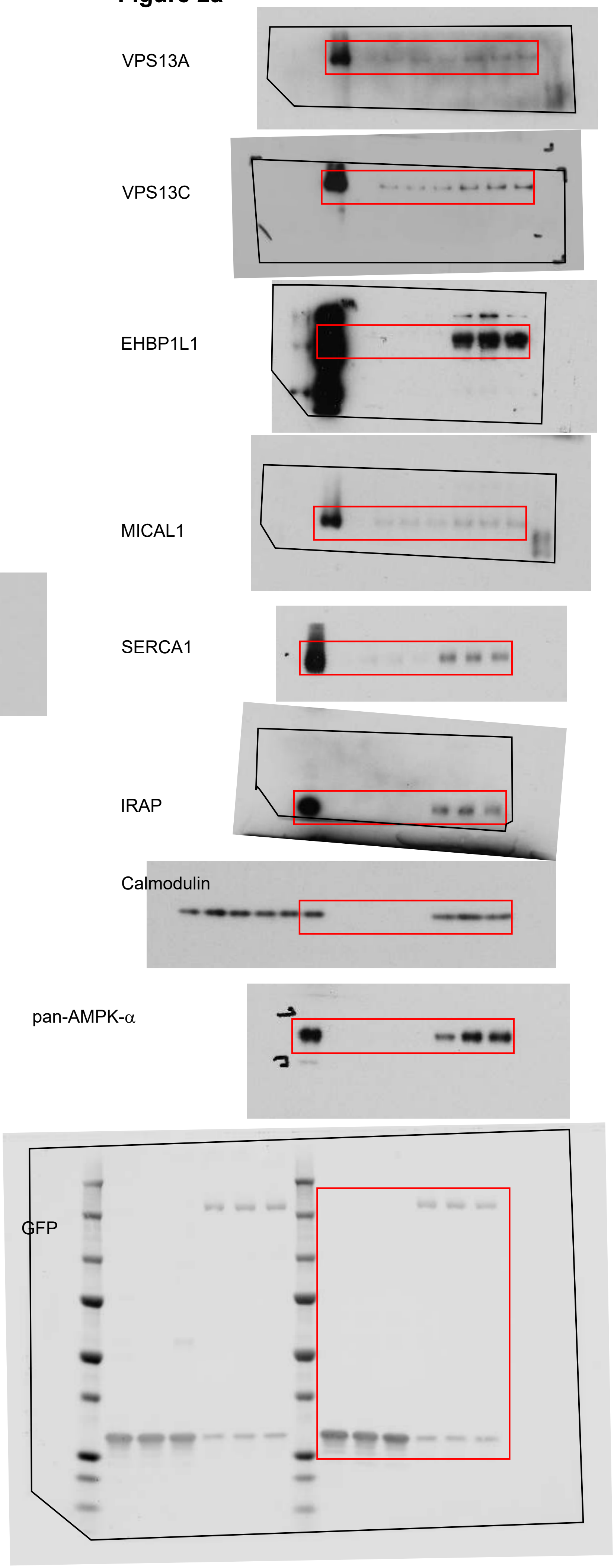

Figure 2c

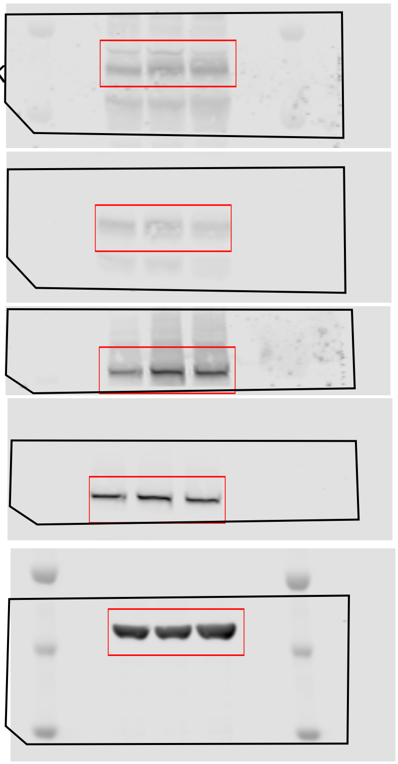

pThr^{172} AMPK

pan-AMPK-α

pSer^{79}ACC

ACC

Actin

Figure 3a

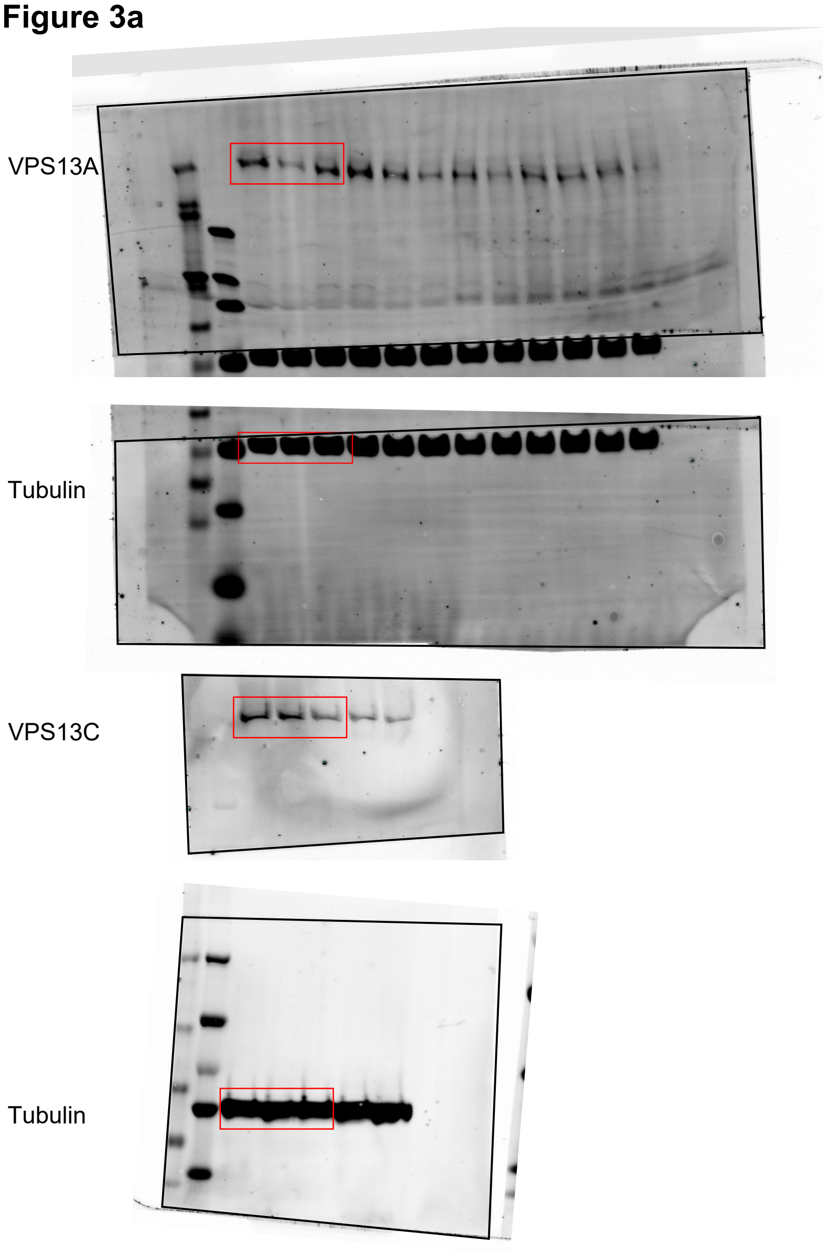

VPS13A

Tubulin

VPS13C

Tubulin

Figure 4e

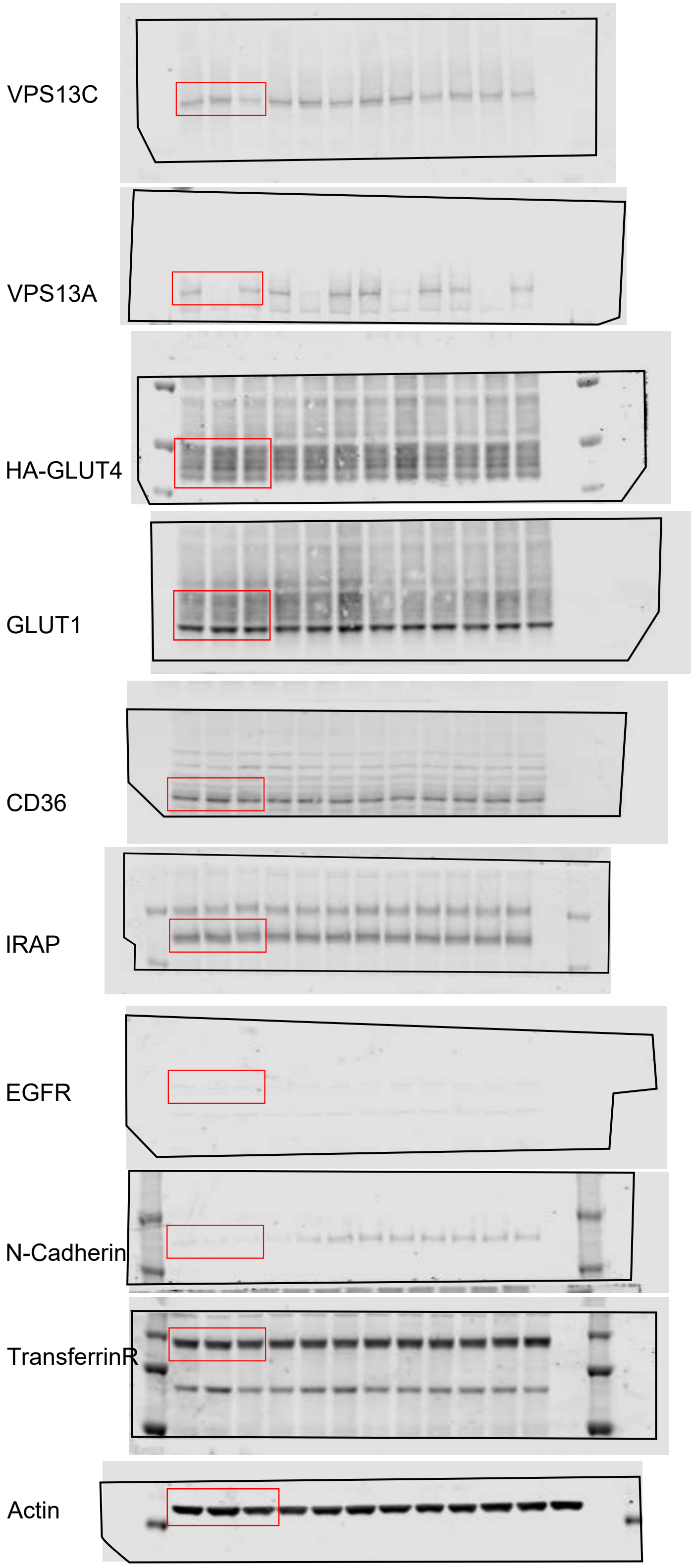

Figure 5c

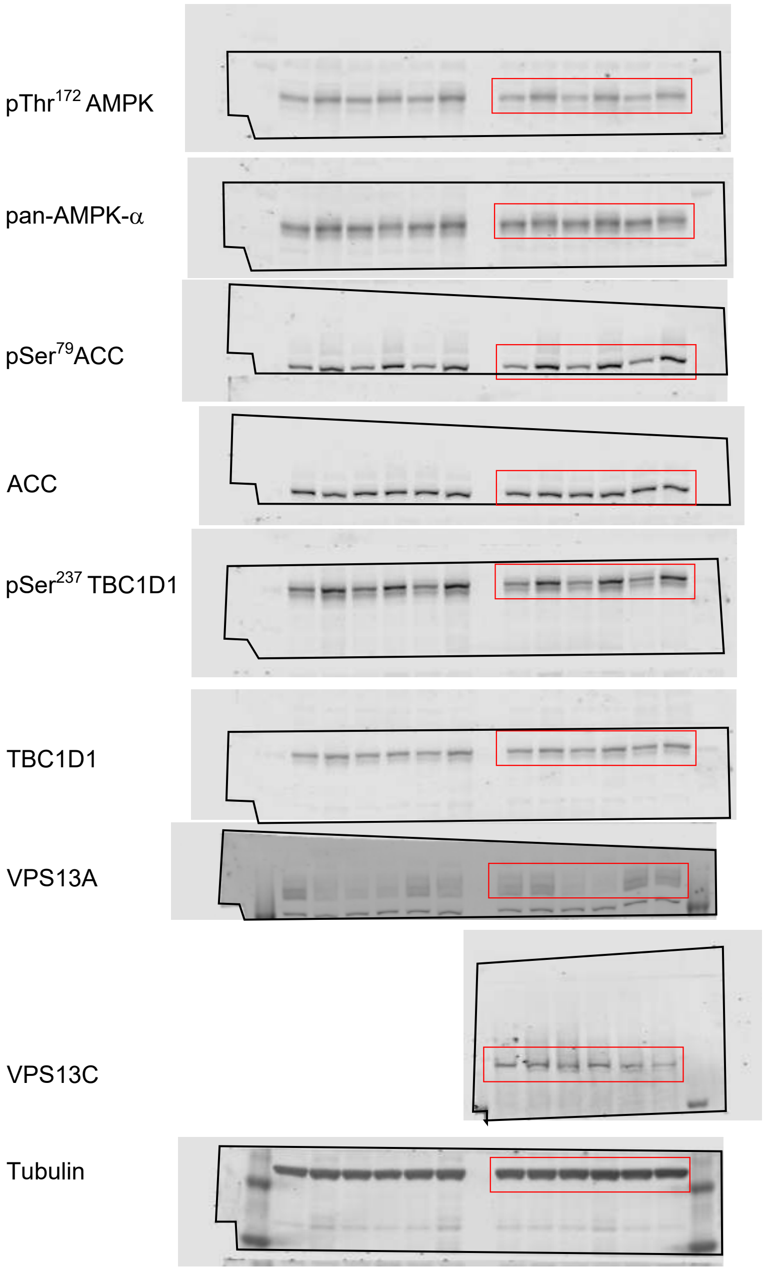

Supplement: Supplementary file 2 — Supplementary file2 [file 41598_2020_74661_MOESM2_ESM.pdf]
